# A vector-free ECG interpretation with P, QRS & T waves as unbalanced transitions between stable configurations of the heart electric field during P-R, S-T & T-P segments

**DOI:** 10.1186/1742-4682-11-10

**Published:** 2014-02-10

**Authors:** Sven Kurbel

**Affiliations:** 1 Department of Physiology, Osijek Medical Faculty, Osijek, Croatia; 2 Osijek University Hospital, Osijek, Croatia

**Keywords:** Electrocardiography, Scalar model, Isoelectric line, P-wave, QRS, T-wave, U-wave, P-R segment, S-T segment, Bundle branch blocks, Ischemic heart disease

## Abstract

Since cell membranes are weak sources of electrostatic fields, this ECG interpretation relies on the analogy between cells and electrets. It is here assumed that cell-bound electric fields unite, reach the body surface and the surrounding space and form the thoracic electric field that consists from two concentric structures: the thoracic wall and the heart. If ECG leads measure differences in electric potentials between skin electrodes, they give scalar values that define position of the electric field center along each lead.

Repolarised heart muscle acts as a stable positive electric source, while depolarized heart muscle produces much weaker negative electric field. During T-P, P-R and S-T segments electric field is stable, only subtle changes are detectable by skin electrodes.

Diastolic electric field forms after ventricular depolarization (T-P segments in the ECG recording). Telediastolic electric field forms after the atria have been depolarized (P-Q segments in the ECG recording). Systolic electric field forms after the ventricular depolarization (S-T segments in the ECG recording).

The three ECG waves (P, QRS and T) can then be described as unbalanced transitions of the heart electric field from one stable configuration to the next and in that process the electric field center is temporarily displaced. In the initial phase of QRS, the rapidly diminishing septal electric field makes measured potentials dependent only on positive charges of the corresponding parts of the left and the right heart that lie within the lead axes. If more positive charges are near the "DOWN" electrode than near the "UP" electrode, a Q wave will be seen, otherwise an R wave is expected. Repolarization of the ventricular muscle is dampened by the early septal muscle repolarization that reduces deflection of T waves. Since the "UP" electrode of most leads is near the usually larger left ventricle muscle, T waves are in these leads positive, although of smaller amplitude and longer duration than the QRS wave in the same lead.

The proposed interpretation is applied to bundle branch blocks, fascicular (hemi-) blocks and changes during heart muscle ischemia.

## Review

Before describing here presented interpretation of the heart electric activity, some introductory remarks seem appropriate. Contemporary physiological and internal medicine textbooks use very similar interpretations of ECG [1-4]. They are all based on the idea that each of the ECG waves (P, QRS and T waves) can be understood as a three-dimensional electric vector that moves in space and time. It is usually assumed that the electric vector loop traces the instantaneous position of the electric wave, as it spreads through the heart muscle. Along the ECG tracing, distinct waves are connected by the “isoelectric line” of near 0 mV, the assumed point of origin of all three wave vectors.

Although this vector-based interpretation has been successfully used in teaching ECG basics for decades, the clinical practice remained focused on the ECG morphology and characteristic wave patterns instead on vectors. This discrepance between the basic ECG interpretation and clinical medicine is well described by a short statement by W. Jonathan Lederer [4]:

“Because the movement of charge (i.e., the spreading wave of electrical activity in the heart) has both a three-dimensional direction and a magnitude, the signal measured on an ECG is a vector. The system that clinicians use to measure the heart's three-dimensional, time-dependent electrical vector is simple to understand and easy to implement, but it can be challenging to interpret.”

Listing several clinically relevant topics not quite suited to the vector interpretation is not difficult. Here are just few examples:

•Textbooks often mention direction of depolarization of the spreading wave: if it is perpendicular to the ECG lead, no voltage is recorded, if it is “approaching” the “positive” (+) electrode, the voltage will be positive, if it is “moving” toward the “negative” (-) electrode, the voltage will be negative.

∘ An example of this interpretation can be found in Boron [4]: “…we can conclude that when the wave of depolarization moves toward the positive lead, there is a positive deflection in the extracellular voltage difference.”. Without detail explanation about the “positive” nature of the approaching depolarization wave, the reader might wrongly conclude that the depolarizing vector direction somehow alters the voltmeter reading, something possibly similar to the Doppler shift in sound or electromagnetic waves coming from a moving object.

•ECG of patients with myocard ischemia show a very peculiar evolution of changes that include S-T elevation, T wave inversion, emergence of Q waves etc. [1-4], most of them are hard to be explained by pure vectors. The most obvious difference is between physiological and clinical interpretation of myocardial ischemia. Guyton & Hall textbook [2] describes it through the idea that after the QRS, in the J point, both ventricles are depolarized and ST segment is the true isoelectric line with no current flowing. Ischemic muscle cannot be adequately repolarised during the T wave and ECG detects the current of injury that offsets the isoelectric line between the T wave and the next QRS. Most cardiology books use the alternative idea that the S-T segment elevation distinguishes patients with myocardial infarction in two differently treated groups based on the ST segment morphology [3]. The patients with the ST elevation on ECG are often abbreviated as cases of STEMI, while others without the elevation are abbreviated as cases of NSTEMI.

•QRS morphology is characteristically altered and prolonged in bundle branch blocks, while in patients with a fascicular block of the left bundle branch (often referred as “hemiblocks”) only the heart electric axis is deviated, while the QRS is not prolonged [3].

•QRS amplitude is routinely used to detect ventricle hypertrophy in our patients, although direct reciprocity of electric amplitude and the heart muscle mass is not clearly present. Explaining higher and prolonged voltage surges recorded during some ventricular premature beats, even in person with normally sized hearts is not easy.

### The reasons behind the quest for an alternative interpretation

During 25 years of teaching the Guyton’s preclinical ECG interpretation to medical students and other profiles of health professionals and working as a clinician, these listed “vector resistant” ECG topics have often made me wander whether the vector interpretation of ECG is fully valid. The turning point was the paper by Harland CJ et al. [5] that describes electrocardiographic monitoring using electric potential sensors placed on wrists without a proper electric contact between sensors and the skin, or even used for remote recording. This means that electric potential sensors measure electric field in the space between the sensors and the actual electric current flowing from well-connected skin electrodes is not necessary for recording.

The initial idea was that sensors might be detecting the electric component of the electromagnetic heart activity, but after reconsidering differences between ECG and MCG data, electromagnetic activity is obviously present mainly during the ECG waves, while “isoelectric” segments induce only very weak magnetic activity, suggesting that electric charges are almost stationary [6]. If the “isoelectric” part of ECG recording is mainly electrostatic by nature, it produces an almost pure electrostatic field, detectable by even remote sensors. Electrodynamic field is generated during the ECG waves that show both electric and magnetic components, detectable by MCG.

An important argument is that any spatial vector is defined by length (magnitude) and direction. Although we are used to consider the spatial position of the “isoelectric” line as a starting point (usually referred as the 0,0,0 point of the three axial vector space) of the heart vector that in each instantaneous moment is directed to another point (defined by x, y, z coordinates). This means that the vector length, or magnitude in mV is a simple three-dimensional diagonal (D) from the starting point to the vector tip:

$$D=\sqrt{\Delta x^{2}+\Delta y^{2}+\Delta z^{2}}$$

This concept would hold true if the momentary heart electric potential during an ECG wave in each millisecond is starts from the point of origin (0,0,0), but this is not the case. Instead of that, the electric field continuously changes its shape and the field center moves in the space. Each millisecond the center takes a new position (x, y, z). In other words, spatial dynamic during an ECG wave can easily be understood as a sequence of still images, quite analogous to individual frames in a motion picture.

Another analogy can be found in membrane potentials usually didactically divided in two: the resting and the action potential. This arbitrary division ignores the simple fact that each millisecond of action potential can be analyzed by the same Goldman equation. In this way, the action is the same as the resting potential, but the membrane permeability changes in time and recalculation of Goldman equation can explain the new potential. In ECG, we are observing an electric field that changes its shape and strength during the heart cycle and in every short moment, the field acts as a stationary field.

### Possible advantages of abandoning the vector-based interpretation

A logical question is which advantages can be gained from developing a vector-free ECG interpretation. There are few possible educational benefits:

•Many students do not accept the vector representation easily, particularly vector projections to frontal and other planes. A simpler interpretation might blunt their initial hesitation to start ECG learning.

•An acceptable new ECG interpretation would need to cover the previously listed clinical entities that do not fit well within the vector model and reduce the gap that exists between preclinicians and clinicians in explaining several ECG topics, like the already mentioned current of injury vs. STEMI.

Possible advantages of using a non vectorial ECG model in studies researching clinical entities are more versatile:

•Contemporary ECG interpretation is focused on the shape and sequence of ECG waves, while the rest of the ECG recording is often labeled as the “isoelectric line”. Perhaps the only important exception is the S-T segment elevation from the other two “isoelectric” segments. The vector-free interpretation might change this “wave-centric” approach into a “panoramic” perspective in which all milliseconds within the heart cycle may contain similar quantity of information.

∘ This approach seems best suited to high resolution three-axial ECG data:

– Data recorded during the three “isoelectric” segments (namely, P-R, S-T and T-P) can be used to detect subtle changes in the position of the electric field that probably result from respiration, heart movements, irregular depolarization or repolarization and possibly vibration of heart walls during diastolic filling (T-P) and systolic ejection (S-T).

– Analogously, data taken during P, QRS and T waves can be used to detect subtle variations, possibly reflecting atrial depolarization (P-wave), ventricular depolarization (QRS) or repolarization (T-wave). Data might reflect the spatial distribution of repolarised and depolarized heart cells within the thoracic cavity.

If consistent information can be extracted from HR ECG data of healthy individuals, the next step would be to correlate then with echocardiography and examine patients with different heart conditions that alter heart anatomy and muscle strength or compliance.

## Basic ideas behind the non vectorial ECG interpretation

The proposed interpretation relies on the analogy between cells and electrets. Cell membrane potential reflects local permeability and concentration gradients of common ions at that instantaneous moment [7,8]. The required concentration gradients across the cell membrane are maintained by Na^+^K^+^ pumps. Due to continuous replenishment of lost ions by new ions that leak from the cell inside, the accumulated positive charges on the outer cell membrane surface behave as virtually membrane attached. This makes living cells weak sources of electrostatic fields. Several authors have put forward the idea that electrostatic fields around cell membrane are similar to electrets [9-11], since an electret is a stable dielectric material with a static electric charge, or with oriented dipole polarization.

Table 1 is intended to give a broader look at similar features of cell membranes and electrets. The main distinction between an electret and the cell membrane is that the membrane is not a permanently polarized dielectric. Instead of that, the membrane polarization is transitory, it depends on ion leakage due to concentration gradients imposed by ion pumping, so it requires energy to be maintained. Cells are more similar to electrostatic machines than electrets, or if we are looking for analogy in magnets, cells are more similar to electromagnets than permanent magnets.

**Table 1:** Comparison of living cells to electrets, electrostatic machines, permanent and electromagnets

**Comparison of field features**	**Magnet and electromagnets**	**Electret and electrostatic machines**	**Living cells**
Stable field maintained without loss of energy	Only in permanent magnets	In electrets due to static bound charges	pH dependent cell protein bound charges
Energy dependent field	Moving electric charges in electromagnets produce magnetic field	In various electrostatic machines temporary electrostatic potentials can be accumulated and discharged	Membrane layered charges depends on ion permeability and ion pumping
Rapid inversion of polarity or rapid depolarization and repolarization	In electromagnets on pulsating or on alternative current	Not easily achieved in electrostatic machines	Electric field is temporary lost and reestablished during action potential
Dipole polarity	Obligatory, there is no magnetic monopole	Usually a dipole configuration that can be reduced to one charge by adequate grounding of one pole	Pericellular electric field is positive or negative, only dipole polarization happens during partial depolarization of excitable cells

Beside that, electrets are similar to permanent magnets in their dipole polarization easily detectable on their surface. Cells with stable membrane potential mimic unit sources of stable electric field that lack the dipole polarization, since cell membrane keeps negative charges hidden inside. A transitory dipole polarization can be found in excitable cells during action potential spreading, when one part of cell membrane is still positive, while the already depolarized part becomes weakly negative.

As it has been briefly described here, excitable cells easily alternate their membrane potential, something that even the electrostatic machines cannot easily do. This unique ability to shift electric potentials in milliseconds is comparable only to electromagnets on a pulsating electric source.

### Electric potentials around the heart muscle cells

If we look at most excitable tissues in more details, their action potentials are very short and the resulting week negative electric fields last only few milliseconds. The main exception is the heart muscle. *Heart muscle cells remains depolarized much longer due to specific shape of the action potential curve [*[1]*,*[2]*]. Beside that, depolarization is synchronized for the whole atrial and ventricular muscle and lasts in hundreds of milliseconds and when the systole is over, normal positive electric fields are quickly reestablished*. Another important feature is that the heart muscle forms a closed shape organ so electric fields around individual cells fuse in a unified heart electric field that changes its strength and shape during the heart cycle. *Repolarised heart muscle acts as a stable positive electric source, while depolarized heart muscle produces much weaker negative electric field, since membrane potential during the heart muscle cell depolarization ranges from 0 to +20 mV, while the repolarised potential is near -90 mV [*[1]*,*[2]*]. This means that the electric field is during depolarization more than four times weaker than in repolarised state*.

During T-P, P-R and S-T segments electric field is stable and only subtle changes can be detected by skin electrodes. These small changes of electric fields can electromagnetically induce only very weak magnetic activity, detectable by MCG [6]. Overall, stationary or slow-moving electric charges mainly produce electrostatic fields with little, or no magnetic actions, so during these three ECG segments (almost 3/4 of the heart cycle), the heart behaves more as a source of an electrostatic than an electrodynamic field. This approach is directly related to the ECG interpretation by RP. Grant in 1950 [12,13]: “*.... studies of the precordial leads are reported which were designed to determine whether these deflections are principally measurements of the electrical field of the heart as a whole or are dominated by the forces from the region of the heart immediately beneath the electrode. It was found that the former was the case, which leads to a simpler and more rational method for interpreting the electrocardiogram than has been available heretofore.*”

### Electrostatic and electromagnetic features of heart electric activity

It is important that any electrostatic field is by definition irrotational, conservative vector field analogous to gravity, possible to be described as the gradient of electrostatic potential, a scalar function. This approach gives us the opportunity to abandon the vector concept when discussing these three “isoelectric” ECG segments.

On the other hand, moving charges produce both magnetic and electric forces, united in the electromagnetic field. Then the three ECG waves (P, QRS and T) can be described as electrodynamic bursts while the heart electric field shifts from one stable configuration to the next. These shifts have already been considered analogous to the waves achieved in a packed football stadium, often referred as "the Mexican wave" [14], that happen when successive groups of spectators briefly stand and return to their usual seated position. The result is a visible wave of standing spectators that travels through the crowd, though individual spectators never move away from their seats. In the heart, waves of depolarized membrane potential seem spreading through neighboring cells, while in fact, membrane permeability to sodium and calcium ions in these cells is just temporarily increased due to action potential. This change of permeability does not require any actual moving charges. Similar to other excitable tissues (skeletal muscles, neurons) action potential among heart muscle cell spreads by influence of the electric fields that affects voltage sensitive channels in the vicinity. The altered polarity spreads due to limited range of electric fields (often referred as electrostatic induction) and almost no actual moving charges are needed. So, instead of trying to imagine actual electric currents moving through the heart muscle, here supported alternative is to consider depolarization as an alteration of the heart electric field due to changed membrane polarity of individual heart muscle cells.

One might argue that electrostatic field could not be maintained since body tissues and fluids are electrically conductive. Keeping in mind that only continuous ion pumping and ion leakages make our cells “pseudoelectrets” is important. Although redistribution of the surrounding ions probably dampens the pericellular electric field, some fraction of the field spreads further due to electrostatic induction of remote structures. The result is that skin electrodes detect brain or heart activity. This means that despite free ion fluxes in body fluids, all cells act as small sources of electric positive charges and these sources fuse and form unified electric fields that surround brain, heart and other organs. Electric fields that emanate around animal bodies are important for prey detection by electroreception found in various aquatic or amphibious predators [15].

Then the heart cycle electric activity can be described as changes in the electric field magnitude and shape during ECG waves, while the field remains nearly stable during the three isoelectric segments of the ECG line.

### Basic assumptions behind the non-vectorial ECG interpretation

The presented interpretation is based on several assumptions:

•The fact that electric potential sensors can record the heart electric activity, even from distance [5], suggests that we should be more concentrated on electric fields that emanate from human body, than on the conventional assumption that ECG measures the electric current that flows between skin electrodes due to a difference in the skin electric potentials.

∘ If we put two electrodes on the opposite sides of the body, as in Frank and other triaxial ECG recordings, each pair of electrodes will measure potential difference even if there is no electric activity since the distribution of electric charges between the electrodes form sources of electric fields that unite into the thoracic electric field.

– This means that any bipolar lead measures the momentary electric field distribution along its axis with temporary negative or positive displacements during ECG waves from the “isoelectric line”. Conventionally, ECG electrodes are labeled as “positive” or “negative”, but in Table 2. “UP” and “DOWN” labels are used as more appropriate to avoid collision with the positive electric field around the repolarised heart muscle and weakly negative electric field around the depolarized muscle:

▪ Bipolar ECG leads: in lead I, the “UP” deflection directs to the left side and “DOWN” to the right side. For leads II and III, the “UP” deflection is toward the heart apex and “DOWN” toward the heart base.

▪ Unipolar ECG leads:

^∗^ For aVL, aVR and aVF, the “UP” deflection points in the direction of the particular extremity, while the “DOWN” deflection points somewhere in the middle of other two extremities.

^∗^ In precordial lads, V1 to V6, the “UP” deflection points more peripherally, to the chest wall, to the chest electrode, while the “DOWN” deflection points toward the central terminal, the referent value that simulates electric potential in the heart center [1-4]. In other words more peripheral charges in the left ventricle wall would give the “UP” deflection, while more central charges, in the right side of the heart would result in “DOWN” deflection.

**Table 2:** The model proposed description of ECG skin electrodes as “UP” and “DOWN” instead of conventional “positive” and “negative” electrodes

**ECG leads**		**DOWN (-)**	**Heart parts along the lead path**			**UP (+)**
Bipolar Einthowen leads	I.	Right arm	Right ventricle wall	Septum	Left ventricle wall	Left arm
	II		Atria	Septum and walls	Apex	Left foot
	III	Left arm				
Unipolar leads from extremities	aVL	Between right arm & left foot	Right ventricle wall	Septum	Left ventricle wall	Left arm
	aVR	Between left arm & left foot	Left ventricle wall	Septum	Atria	Right arm
	aVF	Zpper thoracic aperture, nuchal area	Atria	Septum and walls	Apex	Left foot
Precordial chest leads	V1-2	Central terminal	Deep heart structures	Septum	Atria	Positions on the chest front
	V3-4				Anteroseptal	
	V5-6				Left ventricle wall	
Bipolar triaxiall leads	X	R thoracic wall	Right ventricle wall	Septum	Left ventricle wall	L thoracic wall
	Y	Upper thoracic aperture, nuchal area	Atria	Septum and walls	Apex	Left foot
	Z	Sternal thoracic wall	Ventricle wall	Septum	Ventricle wall	Dorzal thoracic wall

*The basic idea of the presented interpretation is that ECG continuously measures position of the thoracic electric field center*. This field changes its shape, position and strength due to heart electric and pumping electricity, but at any moment, the measured potential difference necessarily reflects only momentary distribution of mainly positive charges in tissues lying between the electrodes.

### Combined electric field of thoracic walls and heart

In most cells some K^+^ ions diffuse from the cell and this surplus of cations on the outer and deficit on the inner membrane side together generate the membrane electric potential. This means that cells from most organs and tissues act as small sources of POSITIVE electric charge (the negative charges remain hidden within each cell).

The highest outwardly positive membrane potentials reach 80 to 90 mV in neurons, skeletal and heart muscle cells [1,2], making them important sources of positive electric potential. Beside that, all these cells develop action potentials. In neurons and skeletal muscles membrane depolarization is very short, just few milliseconds and often occurs in individual cells without much synchronicity. The consequence is that skin electrodes can trace EEG and EMNG electric signals of very low voltage and various frequencies.

The heart muscle electric activity is different [1,2]. The strictly coordinated blood pumping function requires regular electrical activity with synchronized depolarization lasting several hundred milliseconds. The presented interpretation assumes that tiny, cell-bound electric fields fuse into a large electric field that penetrates through body fluid, reaches the body surface and emanates in the surrounding space. After leaving the body, the resulting field obeys the inverse-square law (the field strength is inversely proportional to square of the radius from the source), although due to ionic interactions in body fluids, the electric field spreading through body tissues is probably much more complex.

This all means that the electric potential of a certain point on the body surface or near it is a scalar value, a situation analogous to the temperature distribution through space, or to the pressure distribution in a fluid.

### The “isoelectric” line as electric field oscillations around attractors

The presented interpretation is based on the idea that thoracic tissues produce a positive electric field that emanates from two concentric structures: the thoracic wall and the heart itself. This means that the center of the thoracic electric field changes its position during systole and diastole mainly due to changes in heart muscle polarization, since the outer envelope of electric charges comes from resting thoracic skeletal muscles, sources of an almost unaltered positive electrostatic field.

*The term attractor is here used to describe a setting toward which the thoracic electric field tends to evolve, but without the necessity that the process of changing the thoracic field center position is periodic or chaotic*. Perhaps the best description might be that the ECG data from consecutive heart cycles virtually obey an almost periodic function. This means that ECG data appear to retrace their space trajectories within a given accuracy.

**Figure 1:**
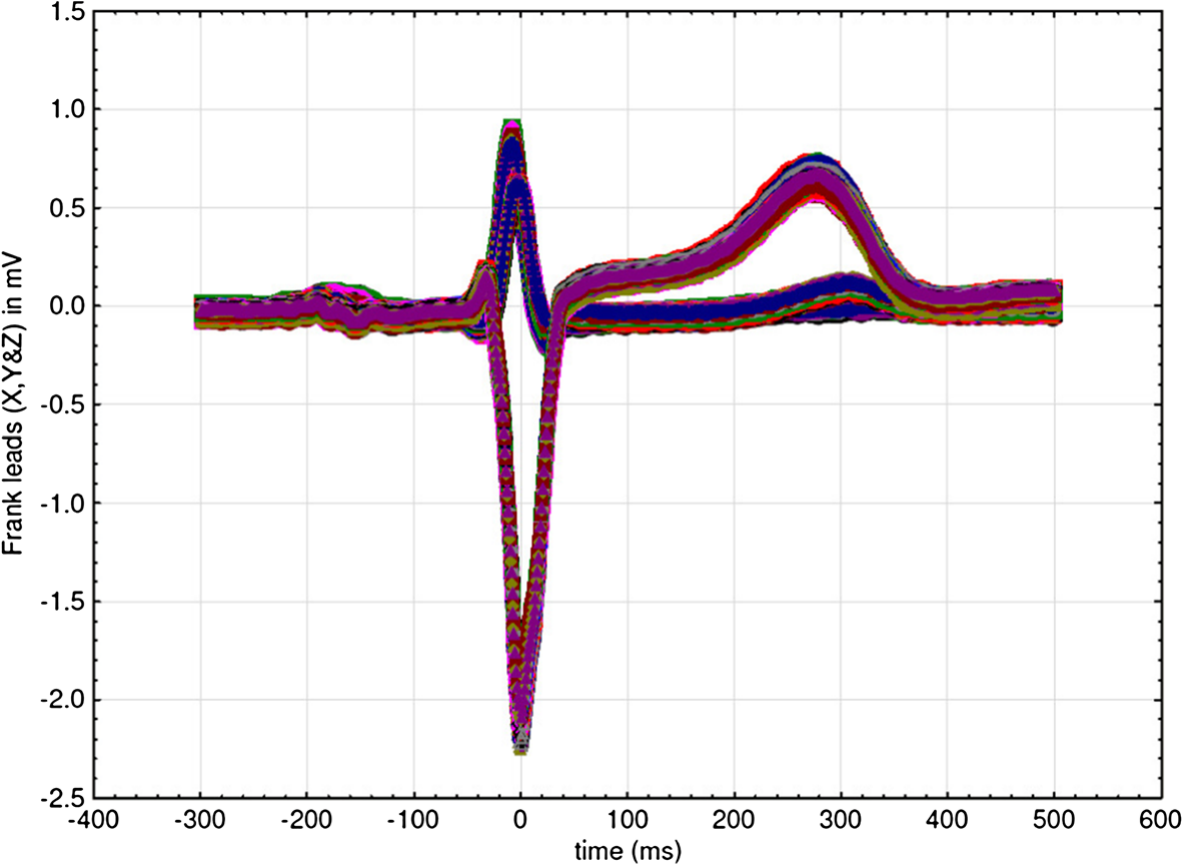
**High resolution (1 KHz sampling rate) triaxial ECG was recorded from a healthy 50 years old male.** Six isoelectric segments of 50 ms were isolated from 100 consecutive heart cycles. Their location was determined from the peak of the R wave (0. ms). These segments are used in Figures 2, 3, 4 and 5: one P-R segment (starting at -125 ms), two S-T segments (ST1 starting at +50 and ST2 starting at +100 ms) and three T-P segments (TP1 starts at +350, TP2 at +450 and TP3 at -250 ms).

Better to illustrate this point, high resolution (1 KHz sampling rate) a triaxial ECG was recorded from a healthy 50 years old male (the author’s own ECG recording). Six isoelectric segments of 50 ms were isolated from 100 consecutive heart cycles (Figure 1). Their location was determined from the peak of the R wave (0. ms): one P-R segment (starting at -125 ms from the R peak), two S-T segments (ST1 starting at +50 and ST2 starting at +100 ms) and three T-P segments (TP1 starts at +350, TP2 at +450 and TP3 at -250 ms).

**Figure 2:**
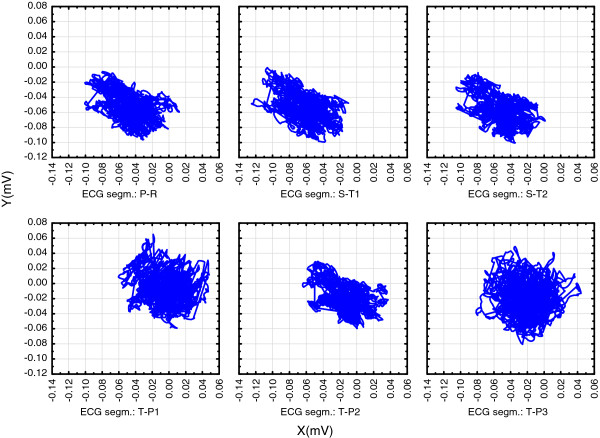
**High resolution (1 KHz sampling rate) triaxial ECG was recorded on a healthy 50 years old males from Figure **1**.** Showing recorded voltages in the frontal (X-Y) plane. In this plane cloud of measured points change its shape but not position, so the center remains almost the same during the entire cycle. This means that in the frontal plane all six segments are isoelectric.

**Figure 3:**
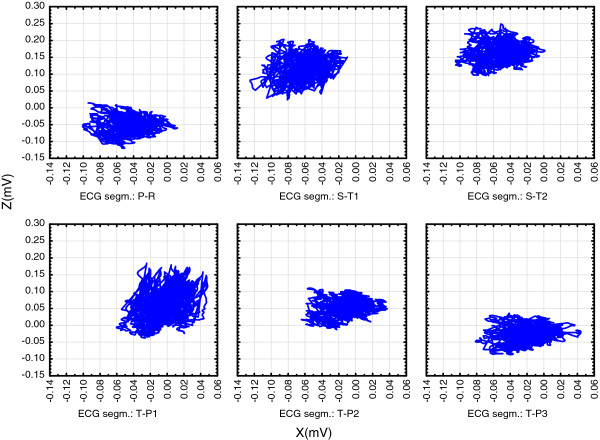
**High resolution (1 KHz sampling rate) triaxial ECG was recorded on a healthy 50 years old male from Figure **1**.** Showing recorded voltages in the horizontal (X-Z) plane. Electric field moves during the cycle: before QRS, in PR it is retrosternal, after QRS it moves dorsally and to the right. T-wave brings it back to the left in TP1 and diastolic feeling moves it back to the retrosternal position in TP3.

**Figure 4:**
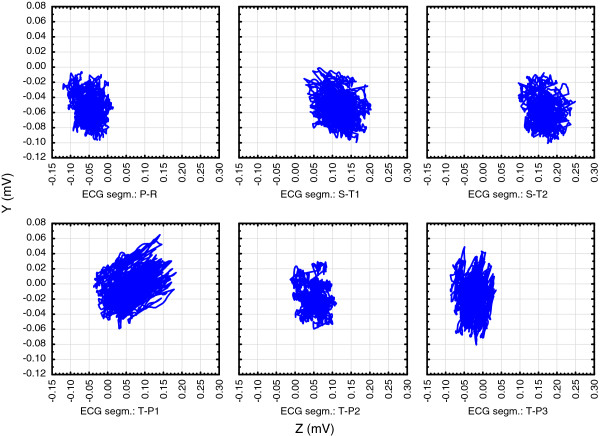
**High resolution (1 KHz sampling rate) triaxial ECG was recorded on a healthy 50 years old male from Figure **1**.** Showing recorded voltages in the sagittal plane. Beside already described movements along the Z axis (Figure 3), diastolic segments (TP1 to TP3) are more caudal than systolic segments.

Figures 2, 3 and 4. show position of these six isoelectric segments in the frontal (Figure 2), horizontal (Figure 3) and sagittal (Figure 4) plane:

•Diastolic electric field forms after ventricular depolarization and stays during most of the diastole (T-P segments in the ECG recording). The field consists of the thoracic wall and completely repolarised heart muscle (shown as TP1 to TP3 in Figures 2, 3 and 4) and its center remains closely around the points in space that can be defined as the diastolic attractor. Any moving of the field center during diastole can be partially attributed to the filling of ventricles with blood that changes the muscle shape and volume.

•Telediastolic electric field forms after the atria have been depolarized (P-Q segments in the ECG recording, shown as PQ in Figures 2, 3 and 4). It consists of the thoracic wall and still repolarised ventricles full of blood. The center also remains closely around the point in space that acts as the telediastolic attractor, normally positioned close to the previously described diastolic attractor.

•Systolic electric field forms after the ventricular depolarization. The field consists of the thoracic wall and recently repolarized atria, while ventricles are depolarized (S-T segments in the ECG recording and shown as ST1 and ST2 in Figures 2, 3 and 4) and the electric field center remains closely around the points in space that act as the systolic attractor. Blood is being expelled during systole, and this changes heart shape and volume.

•Movements of electric field centers in three plains are complex:

∘ Figure 2. shows that in the frontal plane, the clouds of measured points change shape but not position, so attractors of the three “isoelectric” segments remain almost in the same position during the entire cycle. This means that in the frontal plane all six observed segments share similar values as all are belonging to a single “isoelectric” line.

∘ Figure 3. shows that in the horizontal plane clouds of measured points change their shape and position. Attractors take different positions during the cycle: before QRS, in PR the cloud is retrosternal and after QRS it moves dorsally and to the right. T-wave brings the cloud back to the left in TP1 and diastolic feeling moves it back to the retrosternal position in TP3.

∘ Figure 4. shows that in the sagittal plane clouds of measured points change their shape and position. Attractors take different positions during the cycle: Beside already described movements along the Z axis (in Figure 3), diastolic segments (TP1 to TP3) are more caudal than systolic segments, probably due to ventricle expansion.

**Figure 5:**
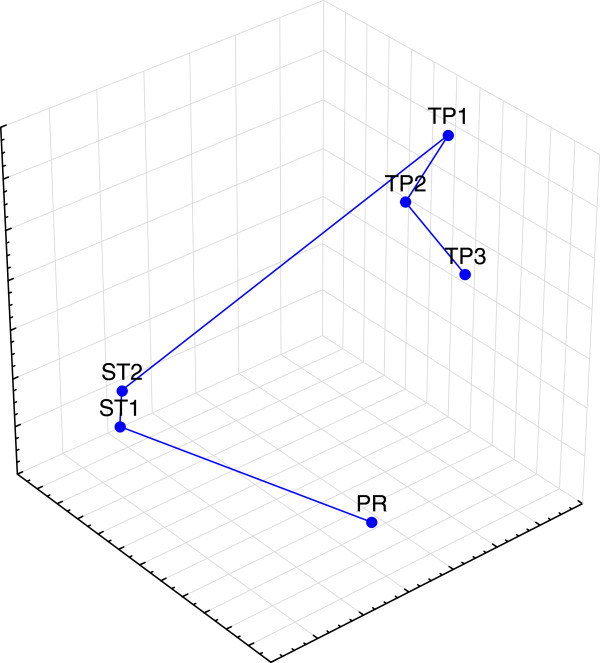
**High resolution (1 KHz sampling rate) triaxial ECG was recorded on a healthy 50 years old male from Figure **1**.** Showing arithmetic means of recorded segments in the 3D space, as substitutes for proposed attractors. Obviously, the electric center moves in space as it is determined by the shape and strength of the heart electric field. P waves happen between TP3 and PR points, QRS between PR and ST1 and T waves between ST2 and TP1. Slight movements from TP1 to TP3 probably reflect diastolic feeling that changes shape of the heart electric field, while differences between ST1 and ST2 probably reflect blood ejection.

∘ Figure 5. shows the arithmetic means of recorded clouds in the 3D space, as substitutes for here proposed attractors. P waves happen between TP3 and PR points, QRS between PR and ST1 and T waves between ST2 and TP1. Slight movements from TP1 to TP3 probably reflect diastolic feeling that changes shape of the heart electric field, while differences between ST1 and ST2 probably reflect blood ejection.

Noting that in the non-vectorial interpretation all ECG waves are simple transition phases between two attractors is important (P wave shows transition between the diastolic and telediastolic attractor, QRS is between the telediastolic and systolic attractor and T wave is between the systolic and diastolic attractors). These transitions are not smooth, symmetric or homogenous, so the electric field center becomes momentarily displaced and this offset causes the three characteristic ECG waves.

In normal individuals, the described attractors are expected to be so near each other in space that in all leads the three correspondent ECG segments form a virtually single “isoelectric line”. In here presented interpretation, the three isoelectric segments are near each other due to concentric anatomical structures and the anchoring effect of the repolarised septal muscle that will be later described in details.

Several conditions that compromise tissue distribution within thorax and/or myocard capacity to depolarize or repolarize can normally offset the systolic attractor from the diastolic and telediastolic attractors. This is evident in the ECG recording as the ST elevation [1-4,16] (often seen in myocardial infarction, Prinzmetal's angina, acute pericarditis, left ventricular aneurysm, pulmonary embolism etc.), or the ST depression (myocardial ischemia, right or left ventricular hypertrophy etc.). So, separation of attractors of “isoelectric” segments can help us explain the nowadays prevailing STEMI versus nonSTEMI concept in clinical practice. It also explains the Guyton’s current of injury in a way that no actuall current exists during diastole. Instead of that, the distribution of heart charges in the diastolic electric field significantly differs from the systolic electric field, due to ischemic areas that are no more able normally to depolarize and repolarize. These alterations in the thoracic field shape and strength displace the systolic attractor from the other two segments.

### The anchoring role of the septal electric field during QRS and T waves

If a bipolar ECG lead measures a difference in electric potentials of two skin electrodes, it gives a scalar value that defines position of the electric field center along that lead: if the “UP” electrode potential prevails we will see an “UP” deflection in the ECG recording *et vice versa*.

Since the potential measured by one of two electrodes come from several electric field sources along the path to the other electrode, we can apply the inverse-square law as a simplified model how proximal and remote charges affect the measured electric potential, as shown in Table 3. We must keep in mind that in our bodies the electric fields probably weaken with distance sooner than the inverse-square law due to ion interactions, protein charges etc., but the well-known inverse-square law seems as a plausible simplification.

**Table 3:** Application of the inverse-square law to the simulation of potentials measured between two opposite ECG lectrodes

**Potential**	**“UP” electrode**		**LV wall**	**Septum**	**RV wall**	**“DOWN” electrode**
The refferent “isoelectric” potential of the P-R segment	Electric field sources	Mass (M)	1.0	0.9	0.7	Distance between electrodes: 1.5 + 0.3 + 0.2 + 1.7 = 3.7
		Potential (P)	50.0	50.0	50.0	
	Distance (Du) from the “UP” electrode		1.5	1.8	2.0	
	Calculated potentials (M × P)/(Du × Du)		22.2	13.9	8.8	
	The P-R potential: 44.9-34.9 = + 10		2.2	1.9	1.7	Distance (Dd) from the “DOWN” electrode
			10.3	12.5	12.1	Calculated potentials (M × P)/(Dd × Dd)
	Total potential on the “UP” electrode		44.9		34.9	Total potential on the “DOWN” electrode
Normal early QRS potential relative to the P-R potential: DOWN deflection makes a Q wave	Electric field sources	Mass (M)	1.0	0.9	0.7	
		Potential (P)	50.0	-10.0	50.0	
	Distance (Du) from the “UP” electrode		1.5	1.8	2.0	
	Calculated potentials (M × P)/(Du × Du)		22.2	-2.8	8.8	
	The absolute potential: 28.2-19.9 = +8.3		2.2	1.9	1.7	Distance (Dd) from the “DOWN” electrode
	Relative to the P-R potential: 8.3-10 = -1.7		10.3	-2.5	12.1	Calculated potentials (M × P)/(Dd × Dd)
	Total potential on the “UP” electrode		28.2		19.9	Total potential on the “DOWN” electrode
Negative potential of the “coronary” Q wave. due to reduced potential of the LV wall	Electric field sources	Mass (M)	1.0	0.9	0.7	
		Potential (P)	10.0	-10.0	50.0	
	Distance (Du) from the “UP” electrode		1.5	1.8	2.0	
	Calculated potentials (M × P)/(Du × Du)		4.4	-2.8	8.8	
	The absolute potential: 10.4-11.7 = -1.3		2.2	1.9	1.7	Distance (Dd) from the “DOWN” electrode
	Relative to the P-R potential: -1.3-10 = -11.3		2.1	-2.5	12.1	Calculated potentials (M × P)/(Dd × Dd)
	Total potential on the “UP” electrode		10.4		11.7	Total potential on the “DOWN” electrode

All unitsa are arbitrary (distance between electrodes is 3.7 units, potential of well repolarised heart muscle is +50 units, potential of ischemic is reduced to positive values <50, while the depolarised muscle is -10 units). The referent “isoelectric P-R segment” is used to explain small, normal Q waves that happen due to early depolarization of the septal muscle. Profound “corronary Q waves” develop due to reduced positive potential of the LV wall caused by only partial repolarization.

For most of 12 ECG leads the septal muscle is the central electric source both for the “UP and the “ DOWN” electrode, although in several leads much more proximal to the “UP” electrode. This means that the repolarised septal muscle is evident for both electrodes, while septal depolarization leaves only lateral ventricle walls as important sources of positive electric field.

### The septal muscle depolarisation and Q waves

Three situations are shown in Table 3;

•If the “UP” electrode, on the left thoracic wall in mid axillary line “sees” first the left ventricle (LV) wall, the septal muscle as the next, more remote source and lastly the right ventricular (RV) wall, it is understandable that for the “UP” electrode, the RV wall is the weakest electric source. The opposite “DOWN” electrode “sees” the sources in the opposite sequence: proximal is the RV wall, then septum and finally the remote LV wall (Table 3).

∘ In individuals with normal anatomy, the “UP” electrode measures higher potential than the “DOWN” electrode and the diastolic potential difference will be slightly higher at the “UP” electrode.

•In the moment of the initial QRS, the septal muscle is depolarized (external charge becomes weakly negative instead of strongly positive), while both LV and RV walls are still repolarised. This means that the septal muscle electrically speaking “vanishes” from the thoracic electric field and then field potential will become relatively more positive (UP) than was the P-R potential, if the LV wall is healthy, thicker and closer to the “UP” electrode than is the RV wall to the “DOWN” electrode. This will result in an R wave, but if the LV wall is damaged, or there is more electric activity in the hypertrophic RV wall, a Q wave will appear.

This interpretation postulates that presence of positively charged septal heart muscle during T-P and P-R segments makes the entire heart electric field more homogenous. Depolarized septal muscle during the initial QRS allows Q to be visible if the repolarised nonseptal heart muscle near the “DOWN” electrode prevails over the similar muscle near the “UP” electrode. Since in our leads almost all “UP” electrodes are monitoring the normally stronger LV wall, any Q usually means that the LV wall electric function is somehow altered and the opposite heart structures prevail in the early phase of QRS.

### The septal muscle repolarisation and the T- wave shape

Repolarization of the ventricular muscle is not initiated through the conductive system so it takes place when muscle cells become ready for depolarization. This process is dampened by the early septal muscle repolarization that reduces amplitude of T waves. The consequence is that since the “UP” electrode of most leads is near the usually larger left ventricle muscle, T waves are in these leads positive, although of smaller amplitude and longer duration than the QRS wave in the same lead.

## Conclusions

As it is already mentioned in the introductory section of this paper, the challenge of the proposed interpretation is to apply it to clinical entities that are not clearly explained by the conventional vectorial description. Tables 4, 5 and 6. Show how the proposed interpretation explains the Q and T wave morphology.

**Table 4:** Comparison of the conventional and the proposed ECG interpretation of the heart cycle phases

**Phases of the ECG cycle**	**The conventional vector interpretation, based on textbook descriptions (1-4)**	**The proposed interpretation based on displacement of the thoracic electric field center**	
		**Description**	**Key events**
T-P segment	Isoelectric line	The diastolic attractor defined by positive charges of atria, ventricles and extracardial thoracic tissues	Most leads transect left ventricle wall, septal muscle and right ventricle wall along a specified line. The repolarised septal muscle makes distribution of charges symmetric and stable
P-wave	Sinoatrial node generates action potential that quickly spreads via internodal fibers. Atrial muscle depolarizes	Displacement of the thoracic electric field center due to diminished charges of the right atria, when both atria become depolarized, the center returns to the telediastolic attractor	SA node initiates atrial depolarization from right to left and from cranial to caudal
P-R segment	isoelectric line	Telediastolic attractor defined by positive charges of ventricles and extracardial thoracic tissues	The repolarised septal muscle makes distribution of charges symmetric and stable
H-wave	AV node depolarizes Hiss bundles before the QRS complex	Signal from the AV node depolarizes the septal muscle	Disappearance of charges within the septum distorts the thoracic electric field and the moving center makes the Hwave.
Q	The septum depolarizes from left to right.	With depolarized septal muscle, there is no anchoring central source of positive charges. In each lead, the electric field center depends on peripheral charges in still polarized ventricular walls.	Q-waves appear if the muscle mass in the wall near the “UP” electrode is reduced (i.e., ischemia) or if the muscle mass near the “DOWN” electrode is increased (i.e., right ventricle hypertrophy).
RS	The anteroseptal region depolarizes first, ventricles depolarize from the endocardium toward the epicardium, spreads from the apex toward the base via Purkinje fibers.	Each lead detects depolarization of left ventricle and right ventricle walls as a rapid displacement of the electric field center.	Maximal displacement is reached when the peripheral part of left ventricle is not yet depolarized. The displacement of the thoracic field center after that quickly diminishes and the center returns to the systolic attractor
S-T segment	The ventricles are fully depolarized.	The thoracic electric field center is back to the systolic attractor defined by positive charges of atria and extracardial thoracic tissues	Both ventricles and the septal muscle are sources of the weak negative charge, with limited influence on the position of the thoracic electric field center.
T-wave	Ventricular repolarization	Emergence of positive ventricular charges displaces the center temporary to the left, caudal and peripheral,	Normally left ventricle wall prevails at the “UP” electrode. Repolarization of the septal muscles makes the emerging positive field stable and almost symmetric,
U wave in precordial leads	Often attributed to repolarisation of papillary muscles or of Purkinje fibers	When both ventricles are repolarised, the thoracic field center returns to the diastolic attractor position.	U wave might reflect rapid diastolic filling of ventricles that temporary changes anatomical position of ventricular walls.

**Table 5:** The proposed interpretation of ECG patterns

**ECG patterns**		**The proposed interpretation based on displacement of the thoracic electric field center**	
		**Description**	**Example**
QRS	qRS	After the septal muscle depolarization, the electric field center moves toward the “DOWN” electrode if the number of positive charges near the “DOWN” electrode is increased (example I), or if the number of positive charges near the “UP” electrode is reduced (example II).	I: Right ventricle hypertrophy
			II: Left ventricle ischemia
	Rs	After the septal muscle depolarization, the electric field center moves toward the “UP” electrode if the number of positive charges near the “UP” electrode is increased, or if the number of positive charges near the “DOWN” electrode is reduced.	I: Normal heart
			II: Left ventricle hypertrophy
	QS	After the septal muscle depolarization, the electric field center moves and remains near the “DOWN” electrode if the number of positive charges near the “UP” electrode is diminished.	Old infarction of the left ventricle wall
	RsR’	Initially the left heart structures near the "UP" electrode remain repolarized, while the deeper right ventricle structures normally depolarize and lose positive charges, thus making the R wave. After the normal right ventricle depolarization via the right bundle branch, left ventricle structures are slowly depolarized through the working myocardium. Slow depolarization further imbalances the thoracic electric field and produces S and R' waves.	Left bundle branch block
T-wave	“Positive”	During the ventricular repolarization, the electric field center moves toward the “UP” electrode if the number of positive charges near the “UP” electrode is larger than near the “DOWN” electrode, the amplitude is reduced by the synchronous septal repolarization	Normal heart
	“Tall”	Altered repolarization of the left ventricle wall is delayed and thus less suppressed by the normal septal repolarization	Ischemia of the left ventricle wall
	“Biphasic”		
	“Negative”	During the ventricular repolarization, the electric field center moves to the “DOWN” electrode if the number of positive charges near the “UP” electrode is smaller than near the “DOWN” electrode.	
U- wave		Due to rapid ventricle feeling in the early diastole dilates repolarized ventricles, making them a rapidly enlarging source of positive electric field. In individuals with good diastolic compliance, this movement of the ventricle walls can transiently displace the electric field center toward the chest electrode.	Often visible in precordial leads

**Table 6:** Comparison of the conventional and the proposed ECG interpretation of the ischemic ECG changes

**The conventional vector interpretation (mainly based on 3, 4, 16)**	**The proposed interpretation based on displacement of the thoracic electric field center**	
	**Description**	**Key events**
	Normal ECG before the onset of plaque rupture	
Hyperacute T wave changes - increased T wave amplitude and width; QT prolongs; some ST segment elevation	altered repolarization of the involved left ventricle wall is not optimally buffered by the still normal repolarization of septal muscle and right ventricle	If walls of both ventricles are synchronous in repolarization, right ventricle and septal muscle reduce the left ventricle dominance. If the left ventricle wall is delayed, the resulting T wave increases
Marked ST elevation with hyperacute T wave changes	altered distribution of ventricular charges in diastole and systole displaces the systolic attractor from the diastolic attractor	any asymmetry in the systolic or in the diastolic ventricular electric field changes the position of the thoracic field center in that heart cycle phase.
Pathologic Q waves appear (necrosis), ST elevation decreases, T waves begin to invert	reduced quantity of repolarised tissue near the “UP” electrode allows the right ventricle structure to prevail during depolarization, when the septal muscle is already depolarized, resulting in Q waves	reduced quantity of tissue able to repolarise near the “UP” electrode allows the septal and right ventricle muscles to prevail and thus Pathologic Q waves and T wave inversion inverse T waves.
Pathologic Q waves and T wave inversion (necrosis with fibrosis)		
Pathologic Q waves, upright T waves (fibrosis)		
Q waves may get smaller or disappear with time	cicatrisation physically reduces the electrically “dead” area, so the surrounding muscle can oppose the prevailing right ventricle and septal muscles	The remaining left ventricle muscle tissue becomes able to repolarise and the new electric balance between two walls and septal muscle is achieved.

Although we often attribute at least the P and QRS waves to the conductive tissue activity, here presented interpretation is based on the idea that almost no electric activity in the conductive tissue is directly evident in the skin surface ECG. Instead of that, ECG traces changes in the position of the thoracic electric field center, so only polarization changes of the large portions of the working heart muscle are detectable.

Although P waves are simultaneous with atrial depolarization, a plausible explanation is that the initial P wave stroke is caused by the displacement of the electric field center toward the heart apex, due to weak negative potential of the atrial muscle. The other part of the P wave can be explained as a hemodynamic change, ventricles are further enlarged due to atrial systole and this moves the electric field center near the initial position, so P-R remains near the T-P segment.

The similar logic is applicable to other ECG waves. Even H wave [5] and U wave [17-19] can be interpreted as temporary imbalances of the thoracic electric field. The H wave can be explained as disturbance of the P-Q (telediastolic) attractor due to partial depolarization of the septal muscle. The U wave, on the other hand, often seen in V3 or V4 lead, may be caused by rapid ventricle feeling. It must be kept in mind that the systolic ventricle volume is normally ½ to 1/3 of the telediastolic volume. When these ventricles of reduced size repolarize at the end of T wave, they become a rapidly enlarging source of positive electric field. In individuals with good diastolic compliance, left ventricle filling can be rapid enough transiently to displace the electric field center toward the V2 or V4 electrode, something happening with the 3^rd^ heart sound.

### Bundle branch blocks

Starting clinical examples with the bundle branch blocks seems appropriate. From the AV node the electrical impulse travels down the Bundle of His and divides into the right and left bundle branches, so bundle branch blocks occur when one branch is interrupted. In leads that show the bundle branch block, T waves are usually deflected opposite the terminal deflection of the QRS complex.

The right bundle branch block is characterized by the widened QRS complex that also shows an extra deflection in the right precordial leads (the V1 lead) [1-4]. The conventional interpretation is that combination R and additional R’ wave reflects the rapid depolarization of the left ventricle followed by the slower depolarization of the right ventricle. Here presented interpretation is that early in the QRS complex, the right heart structures near the “UP” electrode of V1 lead remain repolarized, while the deeper left ventricle structures depolarize. This imbalance with remaining positive charges near the V1 electrode causes the R wave and prevents any q wave. After the normal left ventricle depolarization via the left bundle branch, right ventricle structures are slowly depolarized through the working myocardium. This slow depolarizing process further imbalances the thoracic electric field and produces the sequence of S and R’ waves in the V1 lead. Beside the fact that the RsR’ pattern is visible in the V6 lead, the left bundle branch block seems quite analogous to the RBBB, including the widened QRS complex. Here presented interpretation is that essentialy the same as in the RBBB. QRS starts without any q-wave since the left heart structures near the “UP” electrode of the V6 lead remain repolarized. This imbalance with the depolarized deep structures causes the R wave. After the normal right ventricle depolarization via the right bundle branch, the left ventricle structures are slowly depolarized through the working myocardium. This slow depolarizing process further offsets the thoracic electric field and produces the sequence of S and R’ wave in the V6 lead.

### Fascicular (hemi-) blocks

If we consider bundle branch fascicles, the situation becomes more complex [1-4]. Since the right bundle branch contains one fascicle, the interruption at any position along the branch will result in RBBB. The left bundle branch often subdivides into two fascicles: the left anterior fascicle and the left posterior fascicle. Normal left ventricle depolarization comes from both fascicles and this simultaneous anterior and posterior stimulation result in an intermediary electric axis.

If only one fascicle is interrupted, the left ventricle can still be quickly depolarized from the remaining fascicle. The QRS complex is not widened but the QRS electric axis shows the active fascicle. Left anterior fascicular block causes left axis deviation, while posterior fascicular block causes right deviation.

In the anterior fascicle block, the QRS complex in the lead aVF becomes overall negative (often described as the left deviation of the heart electric axes) and small Q waves can also be seen in leads I and aVL. In the posterior fascicle block, the QRS complexes in the lead I become overall negative (the right axes deviation) while qR complexes are found in leads II, III and aVF [1-4].

Here presented interpretation of fascicular left bundle branch blocks is that we are dealing with an almost normal ventricle depolarization during which the left ventricle depolarization is unidirectional. Due to unidirectional left ventricle depolarization through only one fascicle, the initial part of QRS shows displacement of the electric field center toward the blocked fascicle since this part of the LV remains positive, while the normally stimulated part depolarizes.

In the anterior fascicle block, the electric field center moves dorsally, then left and returns through the anteroseptal segment (anticlockwise from the caudal perspective), In the posterior fascicle block, the electric field center moves in the opposite direction: from anteroseptal to the left and to the dorsal segment (clockwise from the caudal perspective). These depolarization routes deviate the electric axis from the intermediary position (negative QRS in the aVF lead in the anterior hemiblock, or in the I. lead in the posterior hemiblock).

The origin of small q-waves due to anterior hemiblock in leads I and aVL can be explained by the reduced quantity of positive charges near the “UP” electrode of the leads I and aVL moves the electric field center toward the “DOWN” electrode on the right arm. Similar interpretation is for the posterior hemiblock. The left ventricle depolarization through the anterior fascicle in the initial part of QRS reduces quantity of positive charges near the “UP” electrode of the II, III aVF leads that is on the left leg. This moves the electric field center toward the “DOWN” electrodes (separate arms are “DOWN” electrodes for leads II & III, while the combined arm potential is the “DOWN” electrode for the aVF). These changes are visible as small Q wave in these three leads.

It must be noted that when both fascicles are active, their simultaneous actions make the left ventricle depolarization more balanced and any Q wave is normally not visible in the mentioned leads, since depolarization of the septal muscle is not followed by the electric center displacement toward the “DOWN” electrode. Instead of that, the center moves toward the remaining polarized muscle, closer to the “UP” electrode, resulting in the initially “up” deflection, or an R wave in the mentioned leads.

### ECG changes in coronary disease patients

Electrocardiographic changes in patients with acute coronary syndrome show specific evolution. Tables 5 and 6 compare the conventional interpretation (based mainly on [3,4,16]) with the here proposed interpretation based on displacements of the thoracic electric field center.

Often the first change is the emergence of hyperacute T waves that can be interpreted as altered repolarization of the involved left ventricle area that is out of phase with the still normal repolarization of the right ventricle and septal muscle, making the left ventricle repolarization more prominent.

The characteristic ST-segment elevation is interpreted as altered systolic and diastolic distributions of ventricular charges. Any asymmetry in the systolic or in the diastolic ventricular electric field changes the position of the thoracic field center in that heart cycle phase and this offset is visible as the ST-segment elevation. A similar situation of ST elevations often found in cases of left ventricle aneurysm that would make the diastolic distribution asymmetric and different from the systolic distribution.

Pathologic Q wave appearances are conventionally attributed to necrosis of the heart muscle. In the presented interpretation the Q wave mechanism is the same for ischemic and other Q waves. In the early systole, the septal muscle positive electric field disappears due to depolarization and this leaves charges in peripheral ventricular walls to struggle for electric balance. This short phase of imbalanced thoracic electric field is ECG evident as the QRS complex and if positive charges of the right heart side temporary prevail over the reduced positive charges of the corresponding part of the left heart muscle, a Q wave will occur.

This can happen in all three bipolar leads and aVL, aVR and aVF leads. For unipolar precordial leads, if central tissues (mainly the right ventricle) have more positive charges than the peripheral tissue (the frontolateral wall of the left ventricle) Q will emerge in precordial V3 to V6 leads. For the “coronary” Q wave it is proposed that immediately after the septal muscle is depolarized, ischemia of the left ventricle wall reduces quantity of positive charges near the "UP" electrode and thus allows the heart muscle near the “DOWN” electrode to prevail during depolarization.

Similar interpretation is found for the inverted T waves. Left ventricle wall ischemia reduces quantity of tissue able to repolarise near the "UP" electrode. This deficit allows the septal and right ventricle muscles near the “DOWN” electrode to prevail and thus inverse T waves.

Combination of pathologic Q waves and upright T waves is usually attributed to fibrosis. Here proposed interpretation is that the remaining left ventricle muscle tissue recovers and starts repolarization (positive T waves), although remaining unable to prevail over the right ventricle muscle near the “DOWN” electrode during early depolarization.

Q waves may get smaller or disappear with time. This can be interpreted consequently of cicatrisation that physically reduces the electrically "dead" area, so the surrounding muscle can better oppose the still prevailing right ventricle muscle, leading to smaller Q waves.

## Competing interests

The author declares that he has no competing interests.
